# What Do We Know About Alcohol Consumption During Pregnancy and Fetal Alcohol Spectrum Disorder in Nigeria? A Narrative Review

**DOI:** 10.1002/hsr2.73295

**Published:** 2026-10-05

**Authors:** Stephen I. Azumara, Elijah C. Arodiogbu, Penny A. Cook, Raja A. S. Mukherjee, David J. Gilbert

**Affiliations:** ^1^ Department of Clinical Pharmacy and Pharmacy Management University of Nigeria, Nsukka Nsukka Enugu State Nigeria; ^2^ Faculty of Life and Health Sciences Ulster University Coleraine Northern Ireland UK; ^3^ School of Health and Society University of Salford Salford Greater Manchester UK; ^4^ FASD Specialist Behavior Clinic Surrey and Borders Partnership NHS Foundation Trust, Redhill Surrey UK

**Keywords:** alcohol drinking, fetal alcohol spectrum disorders, maternal health, pregnancy, prenatal alcohol exposure

## Abstract

**Background and Aims:**

Alcohol consumption during pregnancy poses notable risks to maternal and foetal health, yet its prevalence and impact in Nigeria remain understudied. This narrative review aims to initiate conversations around the current state of alcohol intake by expectant mothers in Nigeria and Fetal Alcohol Spectrum Disorder (FASD), while providing recommendations to address this public health issue.

**Methods:**

Drawing on existing literature, we highlight the prevalence of alcohol intake by expectant mothers in Nigeria and explore the socio‐cultural factors influencing drinking behaviors during pregnancy. Further to this, we initiate discussions around the impact of maternal alcohol intake with respect to FASD.

**Results:**

Despite limited empirical evidence, available studies suggest that alcohol consumption during pregnancy is not uncommon in Nigeria, although reported prevalence estimates vary across studies and settings. Alcohol use during pregnancy may be influenced by factors such as social norms, lack of awareness, and limited access to prenatal care and education.

**Conclusions:**

Key recommendations include implementing public health campaigns to raise awareness concerning the dangers of alcohol use by expectant mothers, integrating alcohol screening and counseling into routine prenatal care services, strengthening regulations on alcohol advertising and availability, and providing support services for women struggling with alcohol use disorders during pregnancy. By addressing the gaps in knowledge and practice surrounding alcohol consumption during pregnancy, Nigeria can take meaningful steps towards promoting maternal and child health and preventing the lifelong consequences of FASD and related conditions.

## 1 Introduction

Alcohol consumption during pregnancy remains a major cause of adverse maternal and child health outcomes worldwide, including Fetal Alcohol Spectrum Disorders (FASD) [1]. Understanding global patterns of prenatal alcohol exposure provides essential context for interpreting the Nigerian situation. This article presents a narrative overview of selected evidence on alcohol use during pregnancy and FASD, and discusses practical public health strategies that may be implemented within existing healthcare and community systems in Nigeria. Given the dearth of research on this topic in Nigeria, the current article is positioned to initiate discussions in this area. The article is organized thematically and follows previous narrative reviews in this field [2, 3, 4].

## 2 Global Outlook on Alcohol Consumption During Pregnancy

Globally, assessing the rate of alcohol consumption by expectant mothers is challenging for several reasons. Some of the reasons include the absence of precise records documenting drinking habits during pregnancy, inaccuracies in self‐report data, and stigmatization [5, 6]. Nevertheless, studies have aimed to evaluate alcohol consumption in pregnancy, and a systematic review of these in 2017 found that nearly 10% of pregnant people used alcohol during pregnancy globally [7]. Although the studies reveal alcohol consumption patterns across different contexts, they primarily relied on self‐reported data and experimental designs susceptible to recall bias, selection bias, and attrition. Gilligan and colleagues suggest designing questionnaires with standardized beverage measurements to assess alcohol consumption rates, rather than relying on participants' self‐reported estimates [8]. However, methods that depend on participants' recall may be prone to selection bias. Although research has explored biomarkers such as hair analysis for detecting alcohol intake [9, 10], these are not routinely applied in clinical practice for prenatal alcohol exposure. Consequently, most studies rely on self‐reported alcohol use [11, 12, 13], which may underestimate true consumption.

Alcohol consumption during pregnancy has been associated with various sociodemographic factors, although findings are not always consistent across settings [11, 12, 13, 14]. Cannon and colleagues [11] reported higher rates among women who were older, unemployed, unmarried, from non‐Hispanic ethnic minority groups, and had lower educational attainment, findings that align with other studies [12, 13]. In contrast, a British cohort study found alcohol consumption during pregnancy to be more common among women with university‐level education and more affluent backgrounds [14]. These differences suggest that alcohol consumption during pregnancy is not confined to a single socioeconomic group and may be influenced by social, cultural, and contextual factors that vary across populations. Notably, these findings represent associations rather than causal relationships and highlight the need to avoid assumptions or stereotypes regarding alcohol use during pregnancy among particular demographic groups.

Rodriguez and his colleagues found a different social reaction in two Nordic countries that have contrasting societal policies regarding alcohol consumption by expectant mothers, particularly in the context of social adversity [15]. Finnish individuals tend to consume more significant amounts of alcohol when experiencing social adversity, even in light of Finland's conservative stance on alcohol. In contrast, Danish individuals, in comparable situations, consumed less alcohol despite Denmark's permissive stance on drinking. Additionally, research indicates that stressful life events are associated with alcohol consumption [16, 17]. These findings highlight the role of national policies and cultural norms in shaping alcohol consumption, emphasizing the need to consider local context when designing interventions in Nigeria. However, international guidance indicates that no level of alcohol consumption is considered safe during pregnancy, and complete abstinence is recommended to prevent prenatal alcohol exposure [18].

## 3 Alcohol Consumption During Pregnancy in Africa

Africa is an extensive continent with a population exceeding one billion individuals across 54 countries [19]. According to the latest Global Alcohol and Health Status Report [20], people aged ≥ 15 years in the WHO African Region (excluding Tunisia, Egypt, Morocco, Djibouti, Libya, Somalia, and Sudan, classified under the Eastern Mediterranean Region) consume 6.0 L of pure alcohol annually (4.2 L recorded; 1.8 L unrecorded), equivalent to 16.4 mL per day. This consumption corresponds to approximately 13 g/day of ethanol, roughly equivalent to one standard drink per day based on the commonly cited 10 g of ethanol per standard drink; however, definitions of a standard drink vary by country [21].

The World Health Organization (WHO) reports that women in Uganda, Burundi, Rwanda, Gabon, Namibia, and Nigeria are among the highest consumers of pure alcohol per capita (4.4–6.2 L annually) [20]. In contrast, some of the lowest levels of alcohol consumption are reported in Northern Africa, where long‐term abstinence rates exceed 88% [20]. Chad, Namibia, Uganda, and Ethiopia also report high female alcohol consumption (17.7–24.5 L per capita annually), among the highest globally [20].

A systematic review by Popova and colleagues estimated a pooled prevalence of alcohol consumption during pregnancy of 3.4% to 20.5% in Eastern Africa, 6.6% to 14.8% in Western Africa, 2.2% to 12.6% in Central Africa, ranging from 5.7% to 14.2% in Southern Africa, and 4.3% in Northern Africa [22]. Addila and colleagues, using a systematic review, reported a pooled prevalence estimate of alcohol use by expectant mothers in Sub‐Saharan Africa to be 20.8% [23].

Identified risk factors include poor social support and depression [24, 25]. Alcohol use is 1.6 times higher among expectant mothers with depression [23]. Alcohol use is twice as common in unplanned pregnancies [26, 27, 28]. According to these studies, alcohol use during pregnancy was encouraged by peers or friends who also drank. Pregnant individuals who knew that alcohol consumption negatively affects birth outcomes, however, were 64% less likely to drink during their pregnancy than their peers [23].

Despite the WHO global strategy to reduce harmful alcohol use, adopted at the 63rd World Health Assembly and emphasizing prevention among pregnant women and women of reproductive age [29], research across African countries remains limited. Existing studies rely largely on self‐reported data, lack biomarker validation, and inconsistently use validated tools. Consequently, there is a possibility of underreporting, recall bias, or social desirability responses. Additionally, most systematic reviews included only English‐language studies, potentially excluding French publications and introducing reporting bias.

## 4 Alcohol Consumption During Pregnancy in Nigeria: Patterns, Predictors, and Socio‐Cultural Factors

Alcohol consumption during pregnancy is significant in Nigeria, posing potential risks to both parents and unborn children [30]. Ordinioha and Brisibe employed a descriptive cross‐sectional design with an interviewer‐administered questionnaire to evaluate alcohol consumption among 221 pregnant women at the University of Port Harcourt Teaching Hospital in Nigeria. The study assessed the knowledge of women's attitude towards alcohol use and actual alcohol consumption and found that 59.3% consumed alcohol during the index pregnancy [30]. Onwuka and his colleagues, using a cross‐sectional design, determined the rate of alcohol consumption to be 22.6% among 380 pregnant women at the University of Nigeria Teaching Hospital in Nigeria [31]. Martinez and colleagues, using World Health Survey data from adult women across African countries, reported heavy drinking among 38% of current female drinkers in Nigeria [32]. Ordinioha and Brisibe reported high levels of alcohol consumption during pregnancy, with 37.4% of those who consumed alcohol classified as regular drinkers and 25.8% of the total study sample meeting the study definition of binge drinking [30]. Adeoye reported a prevalence of alcohol consumption during pregnancy of 12.7% in the Ibadan Pregnancy Cohort Study [33]. Popova and his colleagues' meta‐analysis combined the results of seven studies to arrive at an overall prevalence of consumption of alcohol during pregnancy of 8.1% (2.5%─15.4%) for Nigeria [22].

Definitions and measurement of alcohol exposure varied across the studies reviewed. Ordinioha and Brisibe defined alcohol consumption as intake of any alcoholic beverage during the index pregnancy, including alcohol consumed as part of native herbal preparations, and defined binge drinking as four or more standard units on a single occasion; one standard drink contained approximately 13 g of ethanol [30]. Onwuka et al. similarly defined alcohol consumption as intake of any alcoholic beverage during the index pregnancy and used approximately 13 g of ethanol per standard drink, but defined binge drinking as four or more standard units on a single occasion within 2 h [31]. Adeoye assessed current alcohol consumption by self‐report and beverage consumption during the preceding 6 weeks; however, the study did not quantitatively measure alcohol consumption and therefore could not assess binge drinking [33]. In contrast, Martinez et al., whose sample comprised adult women rather than specifically pregnant women, defined current drinking as at least one standard drink in the previous 7 days, heavy drinking as 15 or more standard drinks during the previous 7 days, and risky single‐occasion drinking as five or more standard drinks on at least 1 day during that period; standard drinks contained 8–13 g of ethanol depending on the country [32]. These differences in exposure definitions, standard‐drink quantities, recall periods, study populations, and measurement approaches limit direct comparison of prevalence estimates across studies.

Reported prevalence of alcohol consumption during pregnancy varies across Nigerian settings, with lower rates reported in some northern settings and higher rates in some southern settings [30, 33]. These regional differences may reflect interacting cultural, religious, social, economic, healthcare‐related, and environmental influences rather than any single population characteristic. Methodological differences between studies, including differences in study populations, sampling approaches, and measurement of alcohol consumption, may also contribute to apparent regional variation. The available evidence should therefore be interpreted as reflecting contextual associations rather than inherent differences between religious or demographic groups. Alcohol use during pregnancy in Nigeria is best understood within a biopsychosocial and structural framework in which individual behaviors interact with interpersonal, community, economic, healthcare, cultural, and policy environments [30]. Alcohol regulation and availability vary across Nigerian regions, reflecting differences in local laws, cultural and religious norms, and the social acceptability of alcohol, with some jurisdictions imposing restrictions or prohibitions on alcohol sales [33]. Alcohol is also available through informal outlets, including local drinking establishments and market stalls, where alcoholic beverages may be sold at relatively low prices [30]. Alcohol may also be provided free of charge at social and community events, while widespread alcohol advertising may contribute to environments in which drinking is socially normalized [30]. These factors illustrate how regulatory, commercial, cultural, and community environments may interact with individual circumstances to shape alcohol consumption during pregnancy. Emotional states experienced by pregnant women, ranging from excitement to anxiety, have been associated with drinking behaviors [34]. Psychological factors, including intimate partner violence, may also contribute to alcohol use during pregnancy in Nigeria [35, 36, 37]. Marital distress and lower levels of marital satisfaction have also been associated with alcohol use [33, 34, 38].

Sociodemographic factors such as age, educational attainment, marital status, and household income have been associated with alcohol consumption during pregnancy in Nigeria [33]. In Nigeria, individuals between 26 and 34 years old show the highest prevalence of alcohol consumption during pregnancy, while binge drinking is more common among those aged 18 to 25 [39]. Younger individuals, particularly teenagers, tend to be more involved in prenatal alcohol use, potentially due to unplanned pregnancies and associated stressors [40]. Prepregnancy alcohol use emerges as a significant predictor of alcohol consumption during pregnancy, highlighting the significance of early intervention and support for individuals with a record of alcohol use [33]. According to Onwuka and colleagues, individuals with less than a tertiary education were significantly more prone to consume alcohol while pregnant. They suggested that limited knowledge or inadequate education might contribute to this behavior, as those with lower education levels may be less knowledgeable about the dangers of consuming alcohol while pregnant. Specifically, 31.1% of individuals with below secondary education reportedly consume alcohol during pregnancy, compared with 19.3% of those with tertiary education [31]. These associations may also reflect broader barriers, including differences in health literacy and access to healthcare services. Similarly, Adeoye reported that the prevalence of alcohol consumption during pregnancy was 16.33% among individuals with primary education, 12.9% among those with secondary education, and 12.46% among those with tertiary education [33]. Taken together, the available evidence suggests that alcohol consumption during pregnancy in Nigeria cannot be adequately explained by a single cultural, religious, or demographic characteristic. Rather, drinking behaviors appear to occur within interacting individual, interpersonal, social, healthcare, economic, cultural, and regulatory contexts. Prepregnancy alcohol use, psychological and relationship circumstances, socioeconomic conditions, health literacy, access to healthcare and information, alcohol availability and affordability, regulatory environments, and prevailing social and cultural norms may collectively shape alcohol use during pregnancy.

## 5 Effects of Alcohol Consumption on Pregnancy

Alcohol, recognized as a teratogen, can adversely affect foetal development when consumed during pregnancy [1, 41, 42]. It may result in a range of developmental, physical, and health‐related challenges [43, 44, 45, 46]. The severity and complexity of these effects depend upon the dosage, frequency, and timing of alcohol exposure [47, 48, 49]. For instance, alcohol exposure can affect the brain at any stage of pregnancy. However, significant structural changes, such as structural brain changes and characteristic facial differences, are more likely during the first trimester compared with developmental changes later [50, 51, 52], although such physical features are relatively uncommon in clinical practice.

In addition to the quantity and timing of alcohol exposure, there is a wide range of factors that may influence developmental outcomes, such as maternal nutrition [45, 53], smoking [54], and genetic predisposition of the mother and foetus [55]. Furthermore, it is often difficult to distinguish the effects of prenatal alcohol exposure from those associated with adverse childhood experiences, genetics, trauma, family history, and other environmental influences, which may interact to influence developmental outcomes. Therefore, developmental outcomes following prenatal alcohol exposure should be understood as multifactorial and heterogeneous, reflecting the potential interaction of prenatal alcohol exposure with biological, maternal, genetic, social, and environmental factors. Consequently, developmental difficulties among alcohol‐exposed children should not automatically be attributed solely to prenatal alcohol exposure. This finding suggests that the exact levels of alcohol necessary to cause harm are unknown and vary from individual to individual. Because of this variability in susceptibility to risk, epidemiological studies sometimes do not detect harm at low/moderate levels of alcohol consumption. Systematic reviews investigating the outcome of low to mid‐level alcohol use during pregnancy have found that the evidence to confirm either harm or benefit at this level is inconclusive, although some studies included in the reviews reported birth complications [56, 57, 58] and identified specific fatty acid concentrations (essential for foetal neural development) in individuals who consumed alcohol during pregnancy compared with control groups, enhancing the understanding of alcohol's effects on neural development in those whose intake varied from ‘moderate to heavy' amounts. Due to the potentially severe consequences of prenatal alcohol exposure and the unknown risk for each individual, current expert advice is abstinence from alcohol during pregnancy. However, outcomes may be improved by protective factors such as stable caregiving environments, early identification, and access to appropriate support services.

## 6 Overview of FASD

FASD encompasses various disorders resulting from prenatal alcohol exposure [59]. The spectrum includes four closely related conditions: Alcohol‐related neurodevelopmental disorder (ARND), fetal alcohol syndrome (FAS), Alcohol‐Related Birth Defects (ARBD), and Partial Fetal Alcohol Syndrome (PFAS) [60]. FAS is the most commonly identifiable form in the spectrum [61], although the four conditions described exhibit different degrees of associated impairments. The sentinel facial features associated with FAS are relatively uncommon and are influenced by the timing, frequency, and level of prenatal alcohol exposure. Globally, a systematic review and meta‐analysis estimate that FASD affects more than 7 out of every 1000 births [62]. Over half of the countries analyzed in the study showed an elevated FASD prevalence exceeding 1%, with South Africa reporting the highest (exceeding 100 cases per 1000 births), as corroborated by the meta‐analysis conducted by Roozen and colleagues [63]. In the UK, Lange and colleagues reported an FASD rate of 3.24% through a meta‐analysis [62], while McQuire and colleagues, employing a screening algorithm within a UK birth cohort, identified FASD prevalence of 17% for singly imputed data and 7.2% in complete‐case analysis [64]. Based on an annual cohort of close to 4 million live births in the United States, we can anticipate approximately 144,000 newly diagnosed FASD cases annually, averaging approximately 394 cases per day, based on a midpoint prevalence rate of 3.6% [65]. A recent case ascertainment study using a cross‐sectional design among 6639 children in the United States reported a prevalence rate of 1.1% to 5.0% [66]. An increasing body of evidence from different settings highlights the substantial economic consequences of FASD. For example, a systematic review estimates that the annual expenses associated with caring for individuals diagnosed with FASD in Canada range between CAD$300 million and CAD$6 billion, and in the United States, between USD$150 million and USD$9 billion [67]. In the United Kingdom, FASD also exerts a significant economic burden, as parents of children with FASD are unable to work and claim additional benefits [68]. Moreover, it is estimated that educating these children in care will cost GDP£100 million, with support expenses exceeding GDP£3000 per week [68]. These estimates encompass medical, diagnostic, social services, and correctional costs. Nonetheless, these expenses are underestimated due to the prevalence of undiagnosed or misdiagnosed FASD cases, along with a range of unconsidered factors that contribute to overall FASD‐related costs [69]. The financial strain that FASD places on society may be alleviated by recognizing the specific needs of individuals affected by FASD and by implementing timely interventions and appropriate social policies [70, 71].

## 7 Prevalence of FASD in Africa and Nigeria

The prevalence of FASD in Africa, including in Nigeria, remains understudied, with limited literature available. South Africa stands as an exception, with most research publications on FASD originating from this region. There is a significant knowledge gap regarding the prevalence of this condition across the continent, despite evidence of substantial alcohol use among expectant mothers in Africa [7]. In South Africa, May and colleagues reported an FASD prevalence of approximately 310 per 1000 using population‐based, active case ascertainment among a school‐based cohort that included 213 controls: 2 with FAS, 64 with PFAS, 77 with ARND, and 95 with ARBD [72]. The prevalence of FASD in South Africa is as high as 290 per 1000 (or 29%) in the Winelands area, as reported by Olivier and colleagues, who employed active case ascertainment methods, including anthropometric screening, clinical evaluations, neurodevelopmental assessments of first‐graders, dysmorphology exams, and interviews to estimate alcohol consumption during pregnancy [73]. Epidemiological studies conducted in high‐risk communities in the Western Cape Province documented FASD rates of 17%–23%, with FAS rates among school‐age children ranging from 5.9% to 7.9% [74]. Moreover, a review of studies assessing grade 1 school learners in various South African towns reported FASD rates ranging from 2.9% to 29% [74].

Despite evidence of alcohol consumption during pregnancy in Nigeria, the prevalence and population‐level burden of FASD in the country remain uncertain because direct epidemiological evidence is limited. Modeling based on estimates of alcohol consumption has produced an estimated prevalence of FAS of 14.8 (95% CI: 8.9–21.5) per 10,000 in Africa [7]. However, this modeled African estimate should not be interpreted as a direct estimate of FAS or FASD prevalence in Nigeria. Similarly, prevalence estimates from South Africa provide important regional context but cannot be directly extrapolated to Nigeria because of differences in alcohol exposure patterns, population characteristics, socioeconomic and cultural contexts, healthcare access, diagnostic capacity, and study methods. Therefore, the available evidence indicates a need for further investigation but is insufficient to determine the prevalence or population‐level burden of FASD in Nigeria. Population‐based Nigerian studies using standardized measures of prenatal alcohol exposure and validated approaches to FASD ascertainment are needed to establish prevalence and examine geographical variation.

## 8 Impact of Lack of Knowledge About Alcohol Consumption During Pregnancy on the Health Sector in Nigeria

Despite the well‐documented dangers of prenatal alcohol exposure, there remains a lack of awareness among the Nigerian population, particularly among pregnant women and women of reproductive age. For example, a study by Ordinioha and Brisibe found that only 51.58% of respondents were aware of the harmful effects of alcohol on the foetus. Among those who were informed, the majority (62.29%) cited health professionals as their source of information, while others relied on the internet (18.42%), mass media (7.89%), or friends and spouses (11.40%) [30]. Similarly, Onwuka and colleagues reported that 35.5% of respondents recognized the risks of alcohol consumption during pregnancy, whereas 64.5% were unaware. Sources of awareness in this group included print media (33.3%), health professionals (30.4%), electronic media such as the internet (28.9%), and friends or colleagues (6.7%) [31]. Limited awareness of the risks associated with alcohol consumption during pregnancy may have implications for prevention, early intervention, and maternal and child health services in Nigeria. It poses significant risks to maternal and foetal health, leading to adverse birth outcomes and long‐term developmental impairments [75]. These consequences may result in increased demand for healthcare services related to pregnancy complications and developmental disorders, placing additional pressure on healthcare and necessitating increased resources for prenatal care, neonatal intensive care, and long‐term interventions for affected individuals [76, 77]. The lack of awareness among healthcare providers regarding the hazards linked to alcohol consumption during pregnancy results in missed chances for timely intervention and prevention [78]. Insufficient knowledge and training among healthcare professionals may lead to inadequate screening of alcohol use by expectant mothers or the provision of suitable counseling and support. These deficiencies perpetuate a cycle of negative maternal and foetal consequences, exacerbating the strain on Nigeria's already burdened healthcare system [78].

Moreover, there is a transgenerational effect of prenatal alcohol exposure, with each episode of drinking during pregnancy exposing multiple generations to its hazardous effects [79]. Prenatal alcohol exposure has been correlated with elevated mortality rates among individuals diagnosed with FASD and their siblings, presenting a considerable burden on the healthcare system [80, 81]. Individuals affected by FASD frequently need medical care for physical impairments and mental disorders, along with rehabilitation services, specialized education, and social support [78]. Furthermore, studies highlight the increased risks associated with prenatal alcohol exposure, such as cognitive and behavioral deficits, sudden infant death syndrome, neonatal death, prenatal and postnatal growth deficiencies, spontaneous abortion, stillbirth, as well as preterm delivery [82, 83, 84]. All these impacts may be detrimental to the Nigerian health sector.

## 9 Conclusion and Recommendations

Available studies indicate that alcohol consumption during pregnancy occurs in Nigeria, with some studies reporting relatively high levels in particular settings, including 59.3% reporting alcohol consumption during the index pregnancy and 25.8% meeting the study definition of binge drinking in one southern Nigerian study [30]. However, reported prevalence of prenatal alcohol exposure varies across studies and settings, and evidence of prenatal alcohol exposure should not be interpreted as equivalent to evidence of FASD prevalence. Although studies from other countries and modeled estimates provide important context, direct epidemiological evidence from Nigeria remains limited, and the available evidence is insufficient to reliably estimate the national prevalence or population‐level burden of FASD. Therefore, robust population‐based Nigerian studies using standardized exposure measures and validated FASD ascertainment methods are needed to establish the prevalence and population‐level burden of FASD in Nigeria.

Key recommendations for addressing alcohol consumption during pregnancy in Nigeria encompass a multifaceted approach aimed at raising awareness, enhancing healthcare services, and strengthening regulatory measures. One critical recommendation is the implementation of public health campaigns designed to increase awareness about the risks associated with alcohol intake during pregnancy. These campaigns should be strategically designed to reach women of childbearing age, their families, healthcare providers, and the broader community. By leveraging various communication channels such as community outreach programmes, social media, television, and radio, these campaigns can disseminate accurate information about the consequences of prenatal alcohol exposure and promote the adoption of healthier behaviors. Additionally, ensuring universal access to reliable contraception, particularly hormonal methods, is crucial and aligns with global strategies aimed at reducing unintended pregnancies and preventing prenatal alcohol exposure. This universal access to reliable contraception is especially important because a significant proportion of pregnancies are unplanned. Hormonal contraceptives offer a more consistent means of preventing unintended pregnancies compared with methods depending on behavioral changes, such as condoms or abstinence, thus reducing the risk of FASD.

Furthermore, the incorporation of alcohol screening and counseling into routine prenatal care services is vital for early detection and intervention. Healthcare professionals are pivotal in recognizing women who might be susceptible to alcohol consumption during pregnancy and delivering suitable assistance and counseling. By incorporating standardized screening tools and counseling protocols into prenatal care visits, healthcare professionals can effectively assess alcohol consumption patterns, educate pregnant women about the risks, and offer interventions to reduce or abstain from alcohol use during pregnancy.

In addition to healthcare interventions, strengthening regulations on alcohol advertising and availability is paramount in curbing the prevalence of alcohol intake by expectant mothers. Nigeria should enact and enforce strict regulations to restrict the marketing, promotion, and sale of alcoholic beverages, particularly targeting pregnant women and young adults of childbearing age. This approach may involve implementing restrictions on alcohol advertising in media channels frequented by pregnant women, such as health‐related magazines, prenatal clinics, and maternal health websites.

Women who are pregnant and who are known to be struggling with alcohol addiction should be given targeted specialist support to reduce alcohol consumption. Support services should be made available. These may include counseling, substance abuse treatment programmes, and peer support groups tailored to the unique needs of pregnant women. By offering accessible and culturally sensitive support services, Nigeria can empower women to seek help, address their alcohol use disorders, and make healthier choices for themselves and their babies.

In conclusion, addressing the gaps in knowledge and practice surrounding alcohol consumption during pregnancy in Nigeria requires a comprehensive and coordinated effort from various stakeholders, including healthcare providers, civil society, community organizations, and government agencies. However, the current capacity of the health system to provide multidisciplinary FASD assessments may be limited, particularly in resource‐constrained settings, which could restrict formal diagnosis rates. Strengthening clinician training may represent more feasible initial steps. Furthermore, we recommend conducting prevalence studies that include surveys of pregnant women and, where feasible, biomarker testing such as hair or meconium analysis. While biomarker approaches are not routinely implemented in most international health systems and may pose logistical and cost challenges, they may offer additional research value in selected settings. These efforts may strengthen understanding of prenatal alcohol exposure patterns and, when combined with validated approaches to FASD ascertainment, contribute to more reliable estimates of FASD prevalence and population‐level burden. However, support for children with developmental needs should be guided by individual strengths and needs and should not depend solely on confirmation of prenatal alcohol exposure or a formal FASD diagnosis. By implementing the key recommendations outlined above, Nigeria can take meaningful steps to promote maternal and child health, prevent the lifelong consequences of FASD, and ensure a healthier future for its population.

## Author Contributions

**Stephen I. Azumara:** conceptualization, writing – original draft, writing – review and editing, resources. **Elijah C. Arodiogbu:** conceptualization, resources, writing – review and editing, writing – original draft. **Penny A. Cook:** conceptualization, writing – original draft, writing – review and editing, resources. **Raja A. S. Mukherjee:** conceptualization, writing – original draft, writing – review and editing, resources. **David J. Gilbert:** conceptualization, writing – original draft, writing – review and editing, resources.

## Funding

The authors have nothing to report.

## Ethics Statement

Ethical approval was not required for this study as it is a narrative review. No study participants were involved, and the review solely relies on publicly available literature without direct interaction or data collection from individuals.

## Conflicts of Interest

The authors declare no conflicts of interest.

## Transparency Statement

Stephen I. Azumara affirms that this manuscript is an honest, accurate, and transparent account of the study being reported; that no important aspects of the study have been omitted.

## Data Availability

This research did not generate or analyze new data; the concept of data sharing does not apply to this article.
